# Higher Incidence of Atrial Fibrillation in Left Ventricular-to-Right Atrial Shunt Patients

**DOI:** 10.3389/fphys.2020.580624

**Published:** 2020-12-07

**Authors:** Hongda Chou, Hongxia Chen, Juan Xie, Aiqing Xu, Guanyu Mu, Fei Han, Gary Tse, Guangping Li, Tong Liu, Huaying Fu

**Affiliations:** ^1^Tianjin Key Laboratory of Ionic-Molecular Function of Cardiovascular Disease, Tianjin Institute of Cardiology, Department of Cardiology, Second Hospital of Tianjin Medical University, Tianjin, China; ^2^School of Public Health, Tianjin Medical University, Tianjin, China

**Keywords:** Gerbode defect, left ventricular-to-right atrial shunt, atrial fibrillation, atrial remodeling, echocardiography

## Abstract

**Background:** The possible association between atrial fibrillation (AF) and left ventricular-to-right atrial shunt (LVRAS) has never been reported yet. The present study investigated the incidence of AF in LVRAS.

**Methods:** This was a retrospective study of consecutive patients undergoing echocardiography at a single tertiary center. Clinical data, laboratory results and echocardiography parameters such as right atrial area (RAA), right ventricular end diastolic diameter (RVDD) and left atrial diameter (LAD) were compared between LVRAS group and non-LVRAS patients, and between AF and non-AF patients. Propensity score matching was performed to decrease the effect of confounders. Logistic regression analysis and mediation analysis were used to estimate the relationship between LVRAS and AF.

**Results:** A total of 3,436 patients were included, and the incidence of LVRAS was 1.16% (*n* = 40). The LVRAS group had significantly larger RAA, RVDD and LAD compared with non-LVRAS group. Those who suffered from AF showed larger RAA, RVDD and LAD compared with those who maintained sinus rhythm. Multivariable logistic regression showed that gender (OR: 0.608), age (OR: 1.048), LAD (OR: 1.111), mean pulmonary artery blood pressure (mPAP, OR: 1.023), TR (OR: 2.309) and LVRAS (OR: 12.217) were significant factors for AF. RAA could partially mediate the relationship between LVRAS and AF according to the result of mediation analysis.

**Conclusions:** Our study suggested that LVRAS, TR, LAD, mPAP, age and male were risk factors for AF. RA enlargement might underlie mechanism in the higher incidence of AF in LVRAS patients. These findings should be confirmed in larger prospective studies.

## Introduction

Left ventricular-to-right atrial shunt (LVRAS), also called the Gerbode defect, is a rare form of ventricular septal defect, leading to intracardiac shunting from the left ventricle (LV) to the right atrium (RA) (Bustamante and Cheruku, 2017). Two types of LVRAS has been described, depending on whether the defect was superior or inferior to the tricuspid valve (Ozdogan and Cinar, 2012; Ying and Chen, 2013). The infravalvular type is more common, and usually congenital (Millan et al., 2012), with an indirect shunt between the LV and RA. By contrast, the supravalvular type is usually acquired, with the defect located in the atrioventricular portion of the membranous septum, leading to a direct shunt between LV and RA. Small congenital and acquired shunts are usually asymptomatic (Taskesen et al., 2015). Chronic, asymptomatic or small defects are always managed conservatively (Kelle et al., 2009).

Recently, in our clinical practice, we have observed an increasing incidence of atrial fibrillation (AF) in patients with LVRAS. AF is one of the most common arrhythmias in clinics, resulting in increased risk of stroke, pulmonary embolism, cardiac insufficiency, cardiac and all-cause mortality. The mechanism of AF remains incompletely elucidated but involves both structural and electrophysiological remodeling. However, to date there were few studies concerning the relationship between LVRAS and incidence of AF. The aim of the present case-control study was to clarify the relationship between LVRAS and incidence of AF.

## Methods

### Patients

The study protocol was approved by the Institutional Review Board of the Second Hospital of Tianjin Medical University, and informed consent requirement was waived due to the retrospective nature of the study. We retrospectively reviewed data from patients admitted to the cardiology department of the Second Hospital of Tianjin Medical University between January 1, 2017 and June 30, 2018. Diagnosis of LVRAS was made based on the defect located in the atrioventricular portion of the membranous septum in 2D echocardiography and color flow Doppler showed high velocity systolic flow toward the RA in the parasternal four-chamber view and parasternal short-axis view. The diagnosis of LVRAS was made independently by two imaging-cardiologist who reached agreement on the final diagnostic results. All of the diameters of LVRAS were <5 mm. Diagnosis of AF was made by carefully analyzing past medical history, standard electrocardiograms and Holter recordings.

The exclusion criteria were myocardial infarction, primary cardiomyopathy, myocarditis, atrial compression, moderate to massive pericardial effusion, valvular heart disease, other congenital heart disease, permanent pacemaker implantation, chronic renal failure, hemodialysis, hyperthyroidism, cardiac surgery and patients with missing data.

### Echocardiography

**Figure 1:**
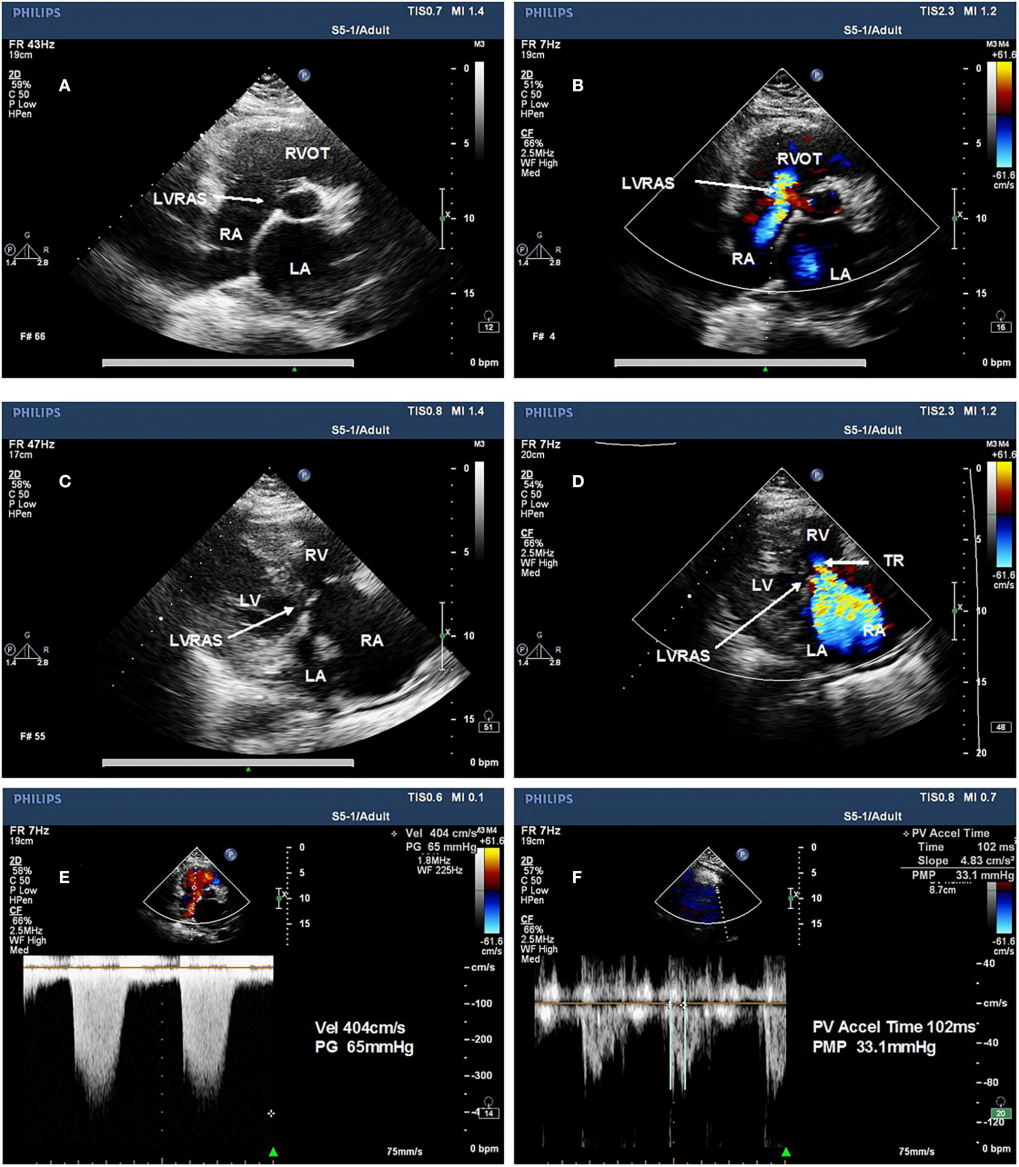
Representative examples of left ventricular-to-right atrial shunt characteristic from a representative patient on TTE imaging. **(A)** small defect in the atrioventricular portion of the interventricular septum in the parasternal short-axis view. **(B)** Left ventricular-to-right atrial shunt in the parasternal short-axis view. **(C)** small defect in the atrioventricular portion of the interventricular septum in the parasternal four chamber view. **(D)** Left ventricular-to-right atrial shunt in the parasternal four-chamber view. **(E)** the pressure gradient of the TR is higher and difference from mean pulmonary pressures and absence of the sign of pulmonary hypertension. **(F)** mean pulmonary pressures of the same patient. LA, left atrium; LV, left ventricle; RA, right atrium; RV, right ventricle.

All patients underwent transthoracic echocardiography (TTE) examination using a commercial ultrasound system (IE33, Philips Healthcare, Inc.). TTE examination was performed using a phased 3D matrix array X5-1 probe. Transesophageal echocardiograph (TEE) examination was conducted with a 3D matrix array probe (X7-2t, carrier frequency 2–7 MHz). Patients underwent 2-dimensional echocardiography with Doppler and tissue Doppler imaging (TDI). Assessments were performed according to recommendations by American Society of Echocardiography (Nagueh et al., 2009). The following parameters were assessed using standard views by TTE: left ventricular end-diastolic diameter (LVDD), left atrial diameter (LAD) and right ventricular end diastolic diameter (RVDD) were measured in the parasternal long axis view; right atrial area (RAA) was measured in the apical four-chamber view; early diastolic LV filling velocities (E peak) and early diastolic septal mitral annular velocity (e' peak) by tissue Doppler imaging were used to calculate the septal E/e' ratio which represents for LV diastolic function; LV ejection fraction (EF) were calculated using Simpson's biplane method in apical four-chamber and two-chamber view; mean pulmonary artery blood pressure (mPAP) was calculated by right ventricular outflow tract flow acceleration time (AT) in the formula: mPAP= 79 – (0.45 × AT). The severity of tricuspid regurgitation (TR) was graded none/trace, mild, moderate or severe according to the semi-quantitative and qualitative assessment by an experienced imaging-cardiologist (Antunes et al., 2008). LVRAS was diagnosed due to the presence of a small defect in the atrioventricular portion of the interventricular septum in the parasternal four-chamber view, and high velocity flow toward the RA in the parasternal four-chamber view and parasternal short-axis view on Doppler imaging by TEE (Figure 1). TEE view used to visualize LVRAS was mid-esophageal four-chamber view.

### Data Collection

Information of gender, age, concomitant diseases such as hypertension, diabetes mellitus (DM), history of smoking, alcohol intake, history of stroke and medication for angiotensin-converting enzyme inhibitors/angiotensin receptor blocker (ACEI/ARB), β-blocker and calcium channel blocker were obtained from the database. The following blood parameters were collected in the database: hemoglobin (HGB), hematocrit (HCT), blood platelet (PLT), platelet distribution width (PDW), albumin (ALB), total bilirubin (TBIL), direct bilirubin (DBIL), indirect bilirubin (IBIL), glucose (Glu), urea, creatinine (Cr), and uric acid (UA).

### Statistical Analysis

Data were presented as means ± standard deviation (SD) for continuous variables and as percentages of the total number of patients for categorical variables. Student's *t*-test was used to compare continuous variables. Qualitative data were presented in numbers (%) and were compared by χ^2^ test. *P* < 0.05 was considered as statistically significant.

Propensity-score matching (PSM) was used to reduce the influence of potential confounders in this observational study. The PSM was performed by matching patients at a 1:1 ratio and without replacement in the two groups by the nearest neighbor technique. The criterion for matching pairs used a caliper width equal to 0.2 of the pooled SD of the logit of propensity score. All analyses were performed with IBM SPSS-24 (IBM, Armonk, NY, USA). PSM was performed with IBM SPSS-24 and Python software (version 1.5.0; JKP IBM SPSS).

Mediation analysis was performed to reveal the indirect effects of RAA on the relationship between LVRAS and AF. It was considered as significant mediator if the bootstrapped values of the 95% confidence interval (CI) did not contain 0 between lower and upper limits (Shigetoh et al., 2019).

All tests were 2-tailed, and *P* < 0.05 was considered significant. Logistic regression analysis was performed to detect which variable provided the strongest influence among all patients enrolled. Meanwhile odds ratios (ORs) and 95% CIs also calculated by logistic regression analysis. Variables that were significant at the *P* < 0.1 level from univariate analysis were enrolled in multivariate logistic regression analysis. Forward stepwise selection was used to select the significant factors to predict AF in the multivariate logistic regression analysis. Then a regression model was created including the variables selected as significant in multivariate logistic regression analysis. The two-tailed *P* < 0.05 in logistic regression analysis was considered as statistically significant.

## Results

### Baseline Characteristics

**Figure 2:**
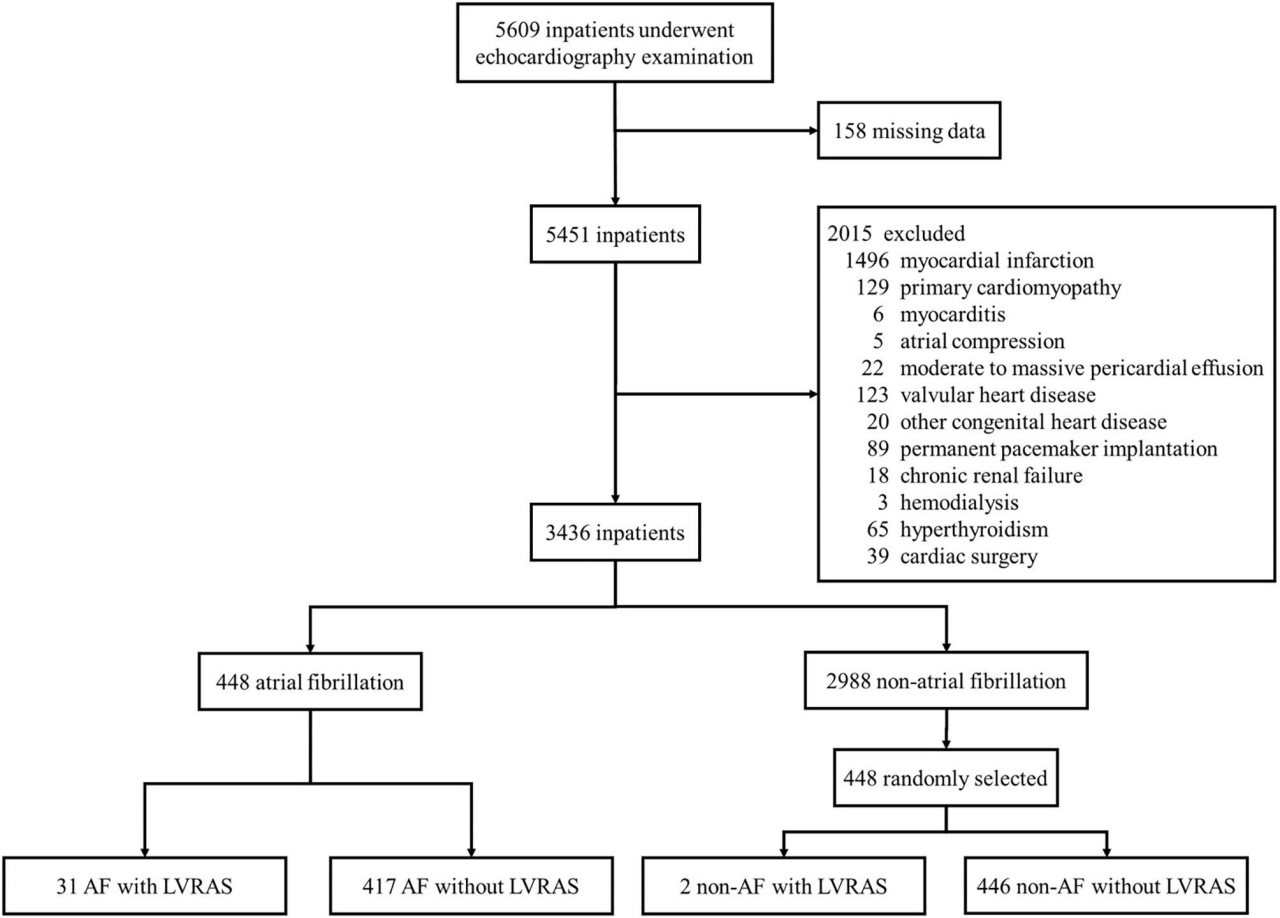
Sample recruitment from cardiology department patients. AF, atrial fibrillation; LVRAS, left ventricular-to-right atrial shunt.

From January 1st, 2017 to June 30, 2018, a total of 5,609 inpatients (mean age, 66.63 [SD, 13.54] years, 48.26% women) in the cardiology department of Second Hospital of Tianjin Medical University underwent echocardiography. Of these, 66 were diagnosed with LVRAS (20 patients diagnosed by TEE). The overall incidence of LVRAS was 1.18%, 50 (75.76%) concomitant AF. There were 3,436 patients remained according to the exclusion criteria: myocardial infarction, primary cardiomyopathy, myocarditis, atrial compressed, moderate to massive pericardial effusion, valvular heart disease, other congenital heart disease, permanent pacemaker implantation, chronic renal failure, hemodialysis, hyperthyroidism, cardiac surgery and patients with missing data. There were 448 AF patients and 40 LVRAS patients enrolled in the study (Figure 2).

### Comparisons Between LVRAS Patients and Non-LVRAS Patients

We compared clinical characteristics, clinical laboratory parameters and echocardiography parameters between LVRAS group (*n* = 40) and non-LVRAS group patients (non-LVRAS group, *n* = 120, 1:3 randomly selected). There was no significant difference in the distributions of gender, hypertension, DM and smoking. Compared with non-LVRAS group patients, LAD (46.06 ± 6.41 mm vs. 37.78 ± 5.83 mm, *P* < 0.001), RAA (22.52 ± 4.97 cm^2^ vs. 13.91 ± 3.48 cm^2^, *P* < 0.001) and RVDD (24.48 ± 4.44 mm vs. 19.85 ± 2.79 mm, *P* < 0.001) were larger in LVRAS group patients. In the LVRAS group, 31 patients (77.5%) combined with AF, 9 patients (22.5%) without AF (Table 1). No significant difference was observed in the incidence of stroke (17.5% vs. 12.5%, *P* = 0.429) between LVRAS group and non-LVRAS group (Table 1).

**Table 1:** Compared LVRAS group and non-LVRAS group.

	**Overall Group**			**PSM Group**		
	**LVRAS**	**Non-LVRAS**	***P-*value**	**LVRAS**	**Non-LVRAS**	***P-*value**
	**(*n* = 40)**	**(*n* = 120)**		**(*n* = 40)**	**(*n* = 40)**	
**Baseline characteristic**						
Male, %	55.0 (22)	48.3 (58)	0.465	55.0 (22)	57.5 (23)	0.822
Age, years	69.08 ± 11.22	65.38 ± 12.55	0.100	69.08 ± 11.22	69.05 ± 15.33	0.993
HTN, %	70.0 (28)	76.7 (92)	0.399	70.0 (28)	72.5 (29)	0.805
DM, %	20.0 (8)	25.8 (31)	0.457	20.0 (8)	15.0 (6)	0.556
Stroke, %	17.5 (7)	12.5 (15)	0.426	17.5 (7)	22.5 (9)	0.576
Smoke, %	12.5 (5)	25.0 (30)	0.098	12.5 (5)	5.0 (2)	0.235
Alcohol intake, %	30.0 (12)	20.0 (24)	0.190	30.0 (12)	12.5 (5)	0.056
AF, %	77.5 (31)	13.3 (16)	<0.001	77.5 (31)	62.5 (25)	0.143
**Echocardiography**						
LAD, mm	46.06 ± 6.41	37.78 ± 5.83	<0.001	46.06 ± 6.41	39.05 ± 7.83	<0.001
LVDD, mm	48.16 ± 4.78	47.66 ± 5.82	0.622	48.16 ± 4.78	46.15 ± 5.40	0.081
EF, %	61.85 ± 7.48	61.88 ± 8.39	0.987	61.85 ± 7.48	59.83 ± 7.90	0.243
RAA, cm^2^	22.52 ± 4.97	13.91 ± 3.48	<0.001	22.52 ± 4.97	16.46 ± 6.24	<0.001
RVDD, mm	24.48 ± 4.44	19.85 ± 2.79	<0.001	24.48 ± 4.44	20.67 ± 3.84	<0.001
E/e	15.55 ± 5.16	13.30 ± 4.48	0.009	15.55 ± 5.16	13.24 ± 4.33	0.034
mPAP, mmHg	28.84 ± 10.01	26.13 ± 11.74	0.198	28.84 ± 10.01	28.69 ± 11.54	0.855
TR, %			<0.001			0.476
None/Trace	20.0 (8)	68.3 (82)		20.0 (8)	27.5 (11)	
Mild	57.5 (23)	29.2 (35)		57.5 (23)	60.0 (24)	
Moderate	15.0 (6)	0.8 (1)		15.0 (6)	5.0 (2)	
Severe	7.5 (3)	1.7 (2)		7.5 (3)	7.5 (3)	
**Clinical laboratory parameters**						
HGB, g/L	135.00 ± 18.15	137.33 ± 15.80	0.443	135.00 ± 18.15	138.65 ± 19.45	0.388
HCT, %	40.97 ± 4.91	41.17 ± 4.32	0.803	40.97 ± 4.91	41.60 ± 5.26	0.583
PLT, 10^9^/L	179.00 ± 43.30	214.12 ± 53.45	<0.001	179.00 ± 43.30	207.13 ± 56.07	0.014
PDW, fL	15.95 ± 0.40	16.03 ± 0.37	0.258	15.95 ± 0.40	16.00 ± 0.48	0.652
ALB, g/L	41.15 ± 3.61	42.68 ± 4.61	0.058	41.15 ± 3.61	41.58 ± 4.51	0.647
TBIL, μmol/L	15.00 ± 6.68	13.12 ± 6.05	0.100	15.00 ± 6.68	15.13 ± 11.68	0.949
DBIL, μmol/L	6.04 ± 4.41	4.20 ± 2.71	0.016	6.04 ± 4.41	5.51 ± 5.05	0.621
IBIL, μmol/L	8.96 ± 3.25	8.92 ± 4.57	0.959	8.96 ± 3.25	9.63 ± 6.96	0.585
GLU, mmol/L	5.54 ± 1.09	6.00 ± 2.07	0.177	5.54 ± 1.09	5.60 ± 1.06	0.801
Urea, mmol/L	6.16 ± 2.63	6.04 ± 2.75	0.809	6.16 ± 2.63	6.08 ± 2.67	0.886
Cr, μmol/L	73.03 ± 21.75	78.25 ± 38.01	0.412	73.03 ± 21.75	70.69 ± 18.35	0.604
UA, μmol/L	360.59 ± 102.25	344.99 ± 117.79	0.456	360.59 ± 102.25	356.22 ± 126.88	0.866
**Medications**						
ACEI/ARB, %	40.0 (16)	47.5 (57)	0.410	40.0 (16)	37.5 (15)	0.818
β-blocker, %	75.0 (30)	22.5 (27)	<0.001	75.0 (30)	25.0 (10)	<0.001
CCB, %	57.5 (23)	40.8 (79)	0.067	57.5 (23)	30.0 (12)	0.013

*Values are mean ± SD or % (n); PSM, propensity-score matching; HTN, hypertension; DM, diabetes mellitus; AF, atrial fibrillation; LAD, left atrial diameter; LVDD, left ventricular end-diastolic diameter; EF%, ejection fraction; RAA, right atrial area; RVDD, right ventricular end diastolic diameter; E/e', the ratio of early diastolic LV filling velocities (E peak) and early mitral annular velocity (e peak); mPAP, mean pulmonary artery blood pressure; TR, tricuspid regurgitation; HGB, hemoglobin; HCT, hematokrit; PLT, blood platelet; PDW, platelet distribution width; ALB, albumin; TBIL, total bilirubin; DBIL, direct bilirubin; IBIL, indirect bilirubin; Cr, creatinine; UA, uric acid; ACEI, angiotensin-converting enzyme inhibitors; ARB, angiotensin receptor blocker; CCB, calcium channel blockers*.

Forty matched pairs of patients were produced according to PSM of the two groups. We matched gender, age, the percentage of hypertension, diabetes, stroke, alcohol intake, smoke, AF and TR. There was no significant difference between the two matched groups for any covariates. Compared with non-LVRAS patients, RAA (22.52 ± 4.97 cm^2^ vs. 16.46 ± 6.24 cm^2^, *P* < 0.001), RVDD (24.48 ± 4.44 mm vs. 20.67 ± 3.84 mm, *P* < 0.001) and LAD (46.06 ± 6.41 mm vs. 39.05 ± 7.83 mm, *P* < 0.001) were significantly larger in LVRAS group.

### Comparisons Between AF Patients and Non-AF Patients

In AF patients (*n* = 448), there were 341 (76.1%) patients associated with hypertension, 132 (29.5%) patients associated with diabetes and 31 (6.9%) patients concomitant LVRAS. Patients without AF (*n* = 448) had lower incidence of LVRAS (2, 0.4%). There was no significant difference in the prevalence of hypertension, diabetes, alcohol intake, smoking status, using of ACEI/ARB and calcium channel blocker in the two groups. Besides, no significant difference was observed in the index of HGB, HCT, PDW and blood Glu in the two groups (*P* > 0.05). Compared with non-AF patients, there were larger RAA (18.34 ± 7.86 cm^2^ vs. 13.79 ± 4.63 cm^2^, *P* < 0.001), LAD (43.47 ± 7.53 mm vs. 37.28 ± 5.44 mm, *P* < 0.001), RVDD (21.53 ± 5.45 mm vs. 19.87 ± 2.66 mm, *P* < 0.001), higher E/e' ratio (15.84 ± 5.85 vs. 13.02 ± 4.41, *P* < 0.001) and relatively lower EF (59.89 ± 8.37% vs. 62.54 ± 6.94%, *P* < 0.001) in AF patients (Table 2).

**Table 2:** Compared AF group and non-AF group.

	**AF**	**Non-AF**	***P*-value**
	**(*n* = 448)**	**(*n* = 448)**	
**Baseline characteristic**			
Male, %	54.0 (242)	47.5 (213)	0.053
Age, years	71.21 ± 11.10	62.98 ± 12.36	<0.001
HTN, %	76.1 (341)	73.0 (327)	0.283
DM, %	29.5 (132)	26.8 (120)	0.373
Stroke, %	20.5 (92)	12.7 (57)	0.002
Alcohol intake, %	17.6 (79)	14.1 (63)	0.143
Smoke, %	26.3 (118)	26.3 (118)	1.000
**Echocardiography**			
LAD, mm	43.47 ± 7.53	37.28 ± 5.44	<0.001
LVDD, mm	48.01 ± 5.71	47.14 ± 5.34	0.893
EF, %	59.89 ± 8.37	62.54 ± 6.94	<0.001
RAA, cm^2^	18.34 ± 7.86	13.79 ± 4.63	<0.001
RVDD, mm	21.53 ± 5.45	19.87 ± 2.66	<0.001
E/e'	15.84 ± 5.85	13.02 ± 4.41	<0.001
mPAP, mmHg	28.82 ± 9.32	24.99 ± 9.52	<0.001
TR			<0.001
None/Trace	35.9 (161)	67.4 (302)	
Mild	53.8 (241)	31.7 (142)	
Moderate	5.8 (26)	0.7 (3)	
Severe	4.5 (20)	0.2 (1)	
LVRAS, %	6.9 (31)	0.4 (2)	<0.001
**Clinical laboratory parameters**			
HGB, g/L	136.39 ± 19.70	135.60 ± 17.76	0.528
HCT, %	41.02 ± 5.50	40.64 ± 4.81	0.273
PLT, 10^9^/L	200.01 ± 62.70	216.76 ± 54.30	<0.001
PDW, fL	16.06 ± 0.40	16.05 ± 0.40	0.563
ALB, g/L	41.45 ± 4.43	42.51 ± 4.07	<0.001
TBIL, μmol/L	15.38 ± 8.67	13.04 ± 6.62	<0.001
DBIL, μmol/L	5.67 ± 4.60	4.19 ± 3.50	<0.001
IBIL, μmol/L	9.70 ± 5.10	8.73 ± 3.98	0.002
Glu, mmol/L	6.06 ± 1.87	5.93 ± 1.70	0.277
Urea, mmol/L	6.67 ± 3.83	5.83 ± 2.72	<0.001
Cr, μmol/L	82.87 ± 47.02	75.23 ± 51.47	0.022
UA, μmol/L	363.96 ± 120.58	326.05 ± 102.43	<0.001
**Medications**			
ACEI/ARB, %	44.4 (199)	39.1 (175)	0.104
β-blocker, %	41.5 (186)	16.7 (75)	<0.001
CCB, %	40.8 (183)	41.7 (187)	0.786

*Values are mean ± SD or % (n); HTN, hypertension; DM, diabetes mellitus; LAD, left atrial diameter; LVDD, left ventricular end-diastolic diameter; EF%, ejection fraction; RAA, right atrial area; RVDD, right ventricular end diastolic diameter; E/e', the ratio of early diastolic LV filling velocities (E peak) and early mitral annular velocity (e peak); mPAP, mean pulmonary artery blood pressure; TR, tricuspid regurgitation; LVRAS, left ventricular-to-right atrial shunt; HGB, hemoglobin; HCT, hematokrit; PLT, blood platelet; PDW, platelet distribution width; ALB, albumin; TBIL, total bilirubin; DBIL, direct bilirubin; IBIL, indirect bilirubin; Cr, creatinine; UA, uric acid; ACEI, angiotensin-converting enzyme inhibitors; ARB, angiotensin receptor blocker; CCB, calcium channel blockers*.

### Comparisons Between AF Patients With LVRAS and Those Without LVRAS

There was no significant difference in rate of hypertension (74.2% vs. 76.3%, *P* = 0.795), DM (19.4% vs. 30.2%, *P* = 0.201) and stroke (16.1% vs. 20.9%, *P* = 0.529) between AF patients with LVRAS and those without LVRAS. Compared with AF patients without LVRAS, RAA (23.58 ± 4.98 cm^2^ vs. 17.95 ± 7.90 cm^2^, *P* < 0.001), RVDD (25.33 ± 4.43 mm vs. 21.25 ± 5.42 mm, *P* < 0.001) and LAD (47.29 ± 6.31 mm vs. 43.18 ± 7.54 mm, *P* = 0.003) were larger in AF concomitant LVRAS patients. There was no significant difference in EF (62.06 ± 6.18% vs. 59.73 ± 8.49%, *P* = 0.134), E/e' (15.82 ± 4.92 vs. 15.84 ± 5.92, *P* = 0.986) and mPAP (27.45 ± 8.87 mmHg vs. 28.94 ± 9.36 mmHg, *P* = 0.393) between the two groups (Table 3).

**Table 3:** Compared AF with LVRAS group and AF without LVRAS group.

	**Overall Group**			**PSM Group**		
	**AF with LVRAS**	**AF without LVRAS**	***P-*value**	**AF with LVRAS**	**AF without LVRAS**	***P-*value**
	**(*n* = 31)**	**(*n* = 417)**		**(*n* = 29)**	**(*n* = 29)**	
**Baseline characteristic**						
Male, %	48.4 (15)	54.4 (227)	0.514	51.7 (15)	62.1 (18)	0.426
Age, years	69.77 ± 9.55	71.32 ± 11.21	0.455	69.03 ± 9.40	68.79 ± 13.57	0.938
HTN, %	74.2 (23)	76.3 (318)	0.795	79.3 (23)	65.5 (19)	0.240
DM, %	19.4 (6)	30.2 (126)	0.201	20.7 (6)	17.2 (5)	0.738
Stroke, %	16.1 (5)	20.9 (87)	0.529	17.2 (5)	13.8 (4)	0.717
Smoke, %	12.9 (4)	27.3 (114)	0.078	13.8 (4)	17.2 (5)	0.717
Alcohol intake, %	35.5 (11)	16.3 (68)	0.007	31.0 (9)	27.6 (8)	0.773
**Echocardiography**						
LAD, mm	47.29 ± 6.31	43.18 ± 7.54	0.003	46.35 ± 5.33	42.08 ± 11.66	0.078
LVDD, mm	47.60 ± 4.82	48.04 ± 5.78	0.681	47.33 ± 4.83	47.88 ± 5.84	0.696
EF, %	62.06 ± 6.18	59.73 ± 8.49	0.134	62.59 ± 6.04	59.90 ± 8.39	0.167
RAA, cm^2^	23.58 ± 4.98	17.95 ± 7.90	<0.001	23.36 ± 4.99	17.32 ± 6.97	<0.001
RVDD, mm	25.33 ± 4.43	21.25 ± 5.42	<0.001	25.25 ± 4.55	21.22 ± 3.55	<0.001
E/e	15.82 ± 4.92	15.84 ± 5.92	0.986	15.73 ± 4.90	13.85 ± 5.09	0.158
mPAP, mmHg	27.45 ± 8.87	28.94 ± 9.36	0.393	26.46 ± 8.13	26.50 ± 7.17	0.982
TR, %			0.004			0.364
None/Trace	19.4 (6)	37.2 (155)		20.7 (6)	24.1 (7)	
Mild	54.8 (17)	53.7 (224)		58.6 (17)	65.5 (19)	
Moderate	19.4 (6)	4.8 (20)		17.2 (5)	3.4 (1)	
Severe	6.5 (2)	4.3 (18)		3.4 (1)	6.9 (2)	
**Clinical laboratory parameters**						
HGB, g/L	137.03 ± 18.32	136.34 ± 19.82	0.852	137.45 ± 18.77	140.69 ± 20.53	0.533
HCT, %	41.53 ± 5.00	40.99 ± 5.53	0.593	41.67 ± 5.10	42.00 ± 5.55	0.814
PLT, 10^9^/L	176.68 ± 40.93	201.77 ± 63.73	0.031	180.09 ± 40.03	196.83 ± 64.21	0.238
PDW, fL	15.95 ± 0.43	16.07 ± 0.40	0.120	15.97 ± 0.43	16.01 ± 0.49	0.692
ALB, g/L	41.09 ± 3.74	41.47 ± 4.49	0.646	41.29 ± 3.74	42.17 ± 4.26	0.405
TBIL, μmol/L	15.61 ± 6.68	15.36 ± 8.81	0.879	15.65 ± 6.84	18.43 ± 13.21	0.319
DBIL, μmol/L	6.25 ± 4.01	5.63 ± 4.64	0.471	6.27 ± 4.13	6.80 ± 5.42	0.674
IBIL, μmol/L	9.36 ± 3.34	9.73 ± 5.21	0.701	9.38 ± 3.38	11.62 ± 8.26	0.184
GLU, mmol/L	5.61 ± 1.09	6.10 ± 1.91	0.167	5.64 ± 1.11	6.19 ± 2.00	0.202
Urea, mmol/L	5.80 ± 2.11	6.74 ± 3.92	0.192	5.89 ± 2.16	6.22 ± 2.78	0.614
Cr, μmol/L	70.75 ± 18.05	83.79 ± 48.41	0.137	71.90 ± 17.95	75.26 ± 29.79	0.605
UA, μmol/L	353.45 ± 91.79	364.72 ± 122.54	0.615	353.99 ± 94.43	332.15 ± 116.31	0.395
**Medications**						
ACEI/ARB, %	41.9 (13)	44.6 (186)	0.773	37.9 (11)	34.5 (10)	0.785
β-blocker, %	83.9 (26)	38.4 (160)	<0.001	82.8 (24)	34.5 (10)	<0.001
CCB, %	58.1 (18)	39.6 (165)	0.043	55.2 (16)	31.0 (9)	0.063

*Values are mean ± SD or % (n); PSM, propensity-score matching; HTN, hypertension; DM, diabetes mellitus; LAD, left atrial diameter; LVDD, left ventricular end-diastolic diameter; EF%, ejection fraction; RAA, right atrial area; RVDD, right ventricular end diastolic diameter; E/e', the ratio of early diastolic LV filling velocities (E peak) and early mitral annular velocity (e peak); mPAP, mean pulmonary artery blood pressure; TR, tricuspid regurgitation; HGB, hemoglobin; HCT, hematokrit; PLT, blood platelet; PDW, platelet distribution width; ALB, albumin; TBIL, total bilirubin; DBIL, direct bilirubin; IBIL, indirect bilirubin; Cr, creatinine; UA, uric acid; ACEI, angiotensin-converting enzyme inhibitors; ARB, angiotensin receptor blocker; CCB, calcium channel blockers*.

PSM of the two groups yielded 29 matched pairs of patients. We matched gender, age, comorbidities such as diabetes, hypertension and stroke, alcohol intake, smoke and TR index. In the two matched groups, no significant difference was observed for any covariates. Compared with AF without LVRAS patients, RAA (23.36 ± 4.99 cm^2^ vs. 17.32 ± 6.97 cm^2^, *P* < 0.001) and RVDD (25.25 ± 4.55 mm vs. 21.22 ± 3.55 mm, *P* < 0.001) were significantly larger in AF with LVRAS group (Table 3).

### Logistic Regression Analysis

Univariate logistic regression analysis showed that gender (OR: 0.772), age (OR: 1.063), LAD (OR: 1.170), EF (OR: 0.954), RAA (OR: 1.170), RVDD (OR: 1.144), E/e' (OR: 1.118), mPAP (OR: 1.045), TR (OR: 3.321), and LVRAS (OR: 16.578) were significantly associated with AF (*P* < 0.1) (Table 4). Then gender, age, LAD, EF, RAA, RVDD, E/e', mPAP and TR were enrolled in the multivariable logistic regression analysis. The multivariable analysis revealed that gender (OR: 0.608), age (OR: 1.048), LAD (OR: 1.111), mPAP (OR: 1.023), TR (OR: 2.309), and LVRAS (OR: 12.217) were significant factors for AF (Table 4).

**Table 4:** Univariate and multivariable logistic regression analysis.

	**Univariate**		***P-*value**	**Multivariable**		***P-*value**
	**β**	**OR (95% CI)**		**β**	**OR (95%CI)**	
Gender	−0.259	0.772 (0.593–1.003)	0.053	−0.498	0.608 (0.430–0.859)	0.005
Age	0.061	1.063 (1.049–1.077)	<0.001	0.047	1.048 (1.032–1.064)	<0.001
HTN	0.165	1.179 (0.873–1.594)	0.283			
DM	0.133	1.142 (0.853–1.528)	0.373			
Alcohol intake	0.269	1.308 (0.912–1.876)	0.144			
LAD	0.157	1.170 (1.140–1.201)	<0.001	0.106	1.111 (1.079–1.145)	<0.001
LVDD	−0.001	0.999 (0.991–1.008)	0.893			
EF	−0.047	0.954 (0.936–0.972)	<0.001			
RAA	0.157	1.170 (1.130–1.211)	<0.001			
RVDD	0.135	1.144 (1.090–1.201)	<0.001			
E/e'	0.111	1.118 (1.085–1.151)	<0.001			
mPAP	0.044	1.045 (1.029–1.061)	<0.001	0.022	1.023 (1.004–1.041)	0.016
TR	1.200	3.321 (2.593–4.254)	<0.001	0.837	2.309 (1.713–3.113)	<0.001
LVRAS	2.808	16.578 (3.943–69.700)	<0.001	2.503	12.217 (2.434–61.334)	<0.001

*HTN, hypertension; DM, diabetes mellitus; LAD, left atrial diameter; LVDD, left ventricular end-diastolic diameter; EF, ejection fraction; RAA, right atrial area; RVDD, right ventricular end diastolic diameter; E/e', the ratio of early diastolic LV filling velocities (E peak) and early mitral annular velocity (e peak); mPAP, mean pulmonary artery blood pressure; TR, tricuspid regurgitation; LVRAS, left ventricular-to-right atrial shunt; β, regression coefficient; OR, odds ratio; CI, confidence intervals*.

### Mediation Analysis

**Figure 3:**
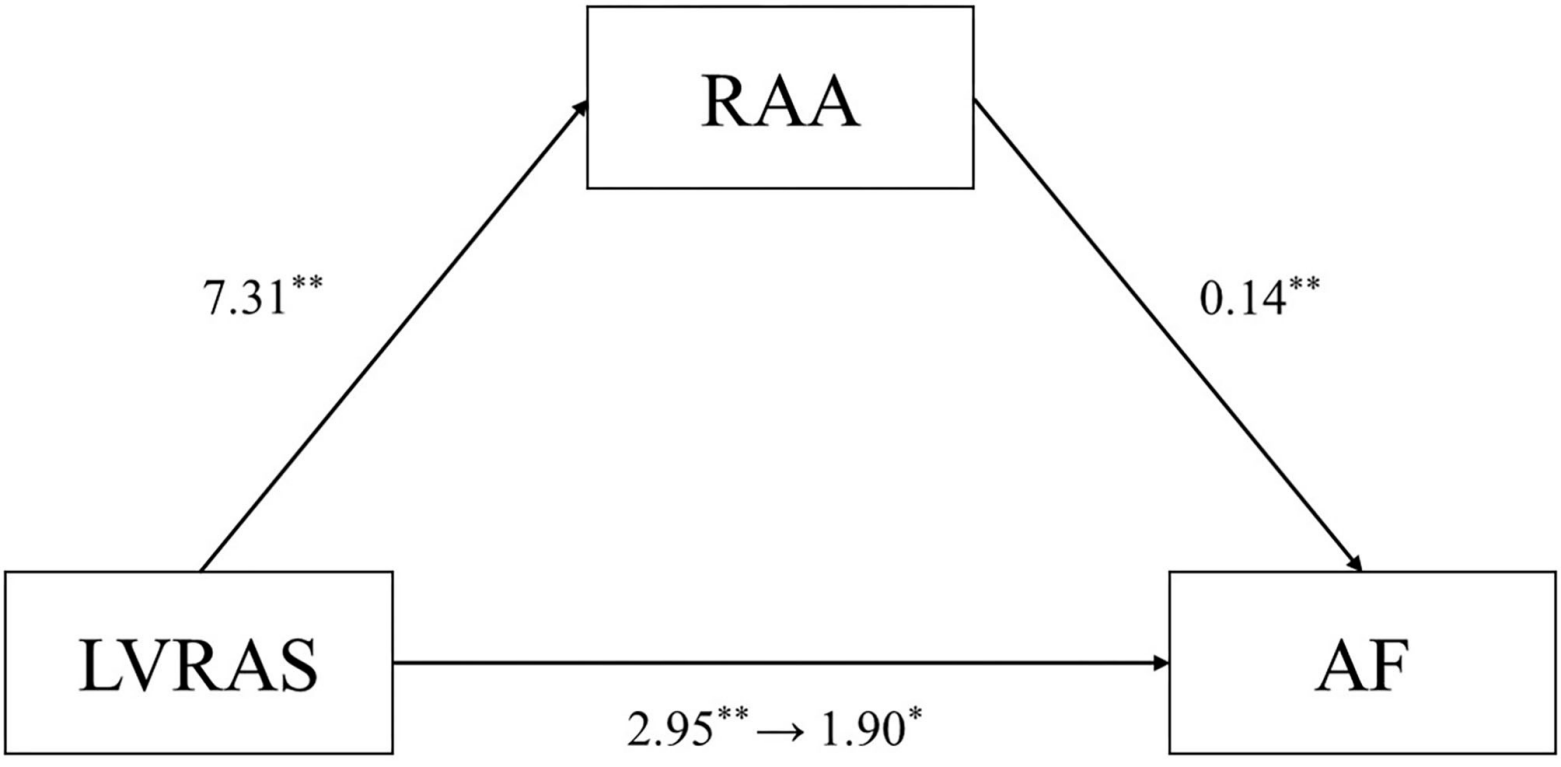
RAA mediates the relationship between LVRAS and AF. LVRAS, left ventricular-to-right atrial shunt; AF, atrial fibrillation; RAA: right atrial area. **P < 0.01 and *P < 0.05.

The mediation analysis result was showed in Figure 3. The direct effect of the hypothesized model was statistically significant for LVRAS (Table 5). Moreover, it showed that the 95% bias corrected (BC) bootstrapped CI for the indirect effects of LVRAS (95% BC bootstrapped CI, 0.336–1.982 with 5,000 resamples) on AF of RAA was significantly different from zero. This result indicated that RAA could partially mediate the relationship between LVRAS and AF.

**Table 5:** Mediation analysis.

**Path**	**Effect**	**β**	**SE**	***P*-value**	**95% BC CI**
a	LVRAS → RAA	7.31	1.47	<0.001	4.418–10.199
b	RAA → AF	0.14	0.02	<0.001	0.109–0.179
c	(direct effect) LVRAS → AF	1.90	0.75	0.012	0.422–3.380
a*b	(indirect effect) LVRAS → AF	1.05	0.43		0.336–1.982

*AF, atrial fibrillation; LVRAS, left ventricular-to-right atrial shunt; RAA, right atrial area; β, regression coefficient; SE, standard error; BC, bias corrected; CI, confidence interval*.

## Discussion

To the best of our knowledge, this was the first study to demonstrate that LVRAS is a significant risk factor for AF development, independently of comorbidities such as diabetes and hypertension, through structural remodeling of the atria. This remodeling was most probably associated with increased blood flow into the RA, leading to higher pressures and dilation of this chamber.

### LVRAS and AF

LVRAS is a type of communication between the LV and the RA (Mousavi et al., 2012). The congenital form is most uncommon and was first described by Gerbode, after whom the condition was subsequently named, in 1958 (Gerbode et al., 1958). Small shunts, whether congenital or acquired, are usually asymptomatic (Taskesen et al., 2015). It was regarded as rare occurrences and not clinically significant (Barclay et al., 1967). Thus, chronic, asymptomatic, or otherwise small defects are managed conservatively (Kelle et al., 2009). However, we had observed an unusually high incidence of AF in patients diagnosed with LVRAS in clinical work. The present study demonstrated the incidence of LVRAS was 1.18%. There were 75.76% of LVRAS patients had concomitant AF. AF has a prevalence of around 1–2% in the general population (Rottlaender et al., 2012). The prevalence of AF in LVRAS is multiple-fold higher. Elevated right atrial pressure and right atrial dilation are two important parameters associated with prevalent AF (Rottlaender et al., 2012). Because of a pressure gradient between the LV and RA, the presence of a LVRAS leaded to increased flow into the RA, and in turn higher right-sided pressures (Saker et al., 2017). This could in turn produce adverse remodeling in the RA that can predispose to AF.

### LVRAS and Atrial Dilatation

The shunt from the LV to the RA resulted in increased right atrial preload because of a significant left-to-right pressure gradient, thus in turn increased blood flow into the right ventricle (RV) (Silbiger et al., 2009). Over time, this leaded to higher pressures initially in the RA and then the RV, with subsequently enlargement of these chambers associated with structural and electrophysiological remodeling. The present study showed RAA, RVDD, and LAD were larger in LVRAS group patients. Since elevated right atrial pressure and right atrial dilatation were risk factors of AF (Rottlaender et al., 2012), LVRAS might predispose to AF via this mechanism.

Enlargement of the atrium always results in structural heterogeneities and regions with increased interstitial fibrosis (Verheule et al., 2003). Previous research has shown that: (1) excessive fibroblast activation due to atrial remodeling can cause increased collagen deposition; (2) non-myocyte such as fibroblasts and myofibroblasts play significant role in mediating abnormal electrophysiology, (3) increased atrial dimension is associated with the change of effective refractory period, which leaded to atrium vulnerable to reentry and maybe the pathological mechanism of AF (Ogunsua et al., 2015; Klesen et al., 2018).

If the shunt between LV and RA is large enough, increased blood volumes will also cause the left heart chambers enlargement (Wu et al., 1994). Left atrial dilatation and dysfunction form a pro-thrombotic status caused by abnormal blood flow and endothelial dysfunction. Left atrial remodeling is an important underlying substrate for AF and stroke (Delgado et al., 2017). It also reported that elevated left atrial pressure played an important role in the development of atrial remodeling in AF patients (Masuda et al., 2018). In the present study, LAD were larger in LVRAS group, which may be due to the left atrium (LA) being stretched when the RA enlarged, or due to the increasing blood flow from right heart to the LA. Thus, the enlargement of RA may be the driving factor for the enlargement of the LA. However, roles of increased LAD in the AF caused by LVRAS needs further study. Moreover, left-sided heart disease with AF was associated with TR progression as well as right-sided heart remodeling. These changes were alleviated by surgical ablation (Wang et al., 2016).

In the present study, the sizes of both atria were larger in AF patients with LVRAS than those in AF patients without LVRAS. In PSM cohort, other risk factors for AF had been obviated, such as the mean age, the percentage of hypertension, the percentage of DM and the rate of alcohol intake. AF patients with LVRAS still had larger RAA while statistically indistinguishable LAD than those without LVRAS. These findings suggested that right atrial remodeling might be an important factor for AF development.

### Characteristic of Echocardiography in LVRAS Group

The appropriate modalities for diagnosing LVRAS are TEE and real-time three-dimensional echocardiography. Using TEE, the most significant findings are RA and RV dilatation, the presence of a small defect in the atrioventricular portion of the interventricular septum in the parasternal four-chamber view, and high velocity flow toward the RA in the parasternal four-chamber view and parasternal short-axis view on Doppler imaging. This has been termed the “bank-shot sign” (Weng et al., 2008), referring to the fact that the curved flow pattern at the LV site of the aneurysm had the appearance of a bank-shot in basketball (Figure 1). As LV systolic pressure is much higher than RA pressure, shunting across LV-RA occurs mainly in systole and maybe trivial, while LV pressure is only slightly higher in diastole (Vallakati et al., 2012). TR could be unstable due to the atypical shunt from LV. Apparent rebound of the flow can be detected: this is due to shunting of blood from the LV to the RA in systole immediately following normal flow from the RA to the RV in diastole. The pressure gradient of the TR is higher and mean pulmonary pressures could be higher, indicating the presence of pulmonary hypertension.

### mPAP and PASP Evaluated by Echocardiography in LVRAS

Pulmonary hypertension is defined as a mPAP ≥ 25 mmHg by right heart catheterization (Galiè et al., 2016). However, echocardiography is widely used (Gorter et al., 2016). Although it is inferior to right heart catheterization in measuring pulmonary pressures, detection rates with echocardiography are similar to those using catheterization (Gorter et al., 2016). In general, PAP is estimated using different methods in echocardiography. The pulmonary artery systolic pressure (PASP) can be calculated with Bernoulli equation, using the peak velocity of the tricuspid regurgitation (TRmax): 4TRmax^2^ + right atrial pressure (RAP). The pulmonary artery diastolic pressure (PADP) can be calculated using the end-diastolic velocity of the pulmonary regurgitation (PR), whereas the mPAP can be estimated using the early peak PR velocity: 4PR^2^ + RAP (Jone and Ivy, 2014). Using pulmonary acceleration time (AT) measured by pulsed-wave Doppler of the pulmonary artery in systole is another way to estimate mPAP, where mPAP = 79 – (0.45 × AT) and mPAP = 90 – (0.62 × AT) in patients with AT <120 ms (Dabestani et al., 1987). In patients with LVRAS, pulmonary artery pressure should be estimated by mPAP rather than PASP, since the reflex flow in the RA not only from the RV but also from the LV via septal defect and PASP may provide an overestimation. Moreover, PASP can only be derived in patients with sufficient TR, and patients with TR were more likely to have higher pulmonary pressures than those without TR (Galiè et al., 2016).

### Right Atrial Fibrillation (RAF) and Right Atrial

Re-entry of excitation wavefronts and focal activity are considered the main mechanisms of AF (Lendeckel and Wolke, 2018). Studies showed spontaneous initiation from the pulmonary vein and left atrial appendage, which were as targets of ablation therapy in vast majority of patients (Haïssaguerre et al., 1998). However, AF is not a pure left atrial disease (Kottkamp, 2019). Since right atrium and left atrium share common muscular fibers, structural remodeling should extend from left atrium to the right atrium (Uemura, 2016). Also, longtime AF could induce histological changes in the right atrial tissues and similarly in left atrial tissues (Vitarelli et al., 2018). However, if the structural remodeling characterized by fibrosis and fatty infiltration are different between patients with RAF and those with left atrial fibrillation (LAF) remains unclear (Lendeckel and Wolke, 2018).

Hiram et al. (2019) showed the right atrial re-entrant activity involving right atrial fibrosis and conduction abnormalities produced a substrate for AF maintenance in a rat model. They provides the clues for the mechanism of AF in pulmonary hypertension or chronic obstructive pulmonary disease could be right atrial enlargement with substantial increase in atrial mass (Medi et al., 2011, 2012). Aksu et al. (2019) also showed RA volume and functions are closely associated with AF development in the postoperative patients. Wen et al. (2017) demonstrated that right atrial diameter (RAD) could be a prediction of arrhythmia recurrence after catheter ablation of AF. It is reported the RA appendage volume index was significantly higher in the RAF patients (Hasebe et al., 2018). Accordingly, the LA appendage/RA appendage volume ratio appears to be much lower in RAF patients than LAF patients, thus providing a potentially useful diagnostic marker that could help to identify AF patients who had no response to pulmonary vein isolation (PVI) (Lendeckel and Wolke, 2018). Hasebe et al. (2016) reported RA ectopic initiation and right-to-left dominant frequency (DF) gradients. The direct relationship between LAD and electromechanical remodeling has been well-established previously (Gaeta et al., 2017). Vitarelli et al. (2018) demonstrated that the atrial septal defect (ASD) patients with right atrial dilatation and dysfunction before device closure was associated with paroxysmal atrial fibrillation (PAF) development. Consistent with findings of the present study, they showed the combination of right atrial volumetric indices with AF risk.

So the right atrium should be an important part of the heart in atrial fibrillation process, and ablation only on the pulmonary vein would be non-responsive in the patients that excitation wavefronts and focal activity were on the right side from the beginning (Medi et al., 2011, 2012; Burnett and Kocheril, 2014; Hayashi et al., 2015; Hasebe et al., 2016, 2018; Uemura, 2016; Gaeta et al., 2017; Wen et al., 2017; Vitarelli et al., 2018; Aksu et al., 2019; Hiram et al., 2019). However, few studies have assessed AF associated with LVRAS and dilated left and right atrium, the mechanism of RAF is still remaining unclear. RA dilation and dyssynchrony caused by LV-RA shunt may lead to electrical remodeling and structural remodeling, which become a basis for AF development (Vitarelli et al., 2018).

On the other hand, chronic atrial dilatation may change atrial electrophysiology. Verheule et al. (2003) demonstrated that chronic left atrial dilatation associated with increased atrial effective refractory period (AERP) and increased AERP dispersion, both of which contributed to the vulnerability of AF. In a clinical case, Morton et al. (2003) also showed that chronic right atrial pressure increasing due to an atrial septal defect caused an increase in AERP. While Suzuki and Takeishi (2012) demonstrated AERP in the aortic constriction (LVH) rats was longer than that in the sham rats regardless of the degree of RA pressure. So the mechanism needed more studies to clarify.

### Predictors of AF in LVRAS

Our results showed the risk factors for AF were LVRAS, TR, LAD, mPAP, age, and gender according to logistic regression analysis. The strongest risk factor was LVRAS. Univariate logistic regression analysis showed that RAA was a significate factor for AF. But in multivariable logistic regression analysis, there was no significant relationship between RAA and AF. The difference between the results may be due to the interaction between LVRAS and RAA. Therefore, mediation analysis was set up to verify this possibility. The result indicated that RAA played a part of role in the relationship between LVRAS and AF. Given that there is RA enlargement in LVRAS, this might represent a mechanistic link to the increased AF occurrence in this content. But this underlie mechanism needed more studies to clarify.

## Limitations

This study had several limitations. The present study was a single-center, retrospective, observational study, and only a small number of patients were enrolled. A major limitation is the small number of cases in the LVRAS group with AF; however, to our knowledge the present study was the first study that involved relatively large cohort of patients with the rare malformation LVRAS. We did not differentiate paroxysmal AF and persistent AF in the study, which may have different underlying mechanisms. Additional confounders associated with LVRAS and AF may not have been detected by our study.

## Conclusions

Our study suggested that LVRAS, TR, LAD, mPAP, age and male were risk factors for AF. RA enlargement might underlie mechanism in the higher incidence of AF in LVRAS patients. The mechanisms underlying atrial electrophysiological remodeling in LVRAS patients remain to be clarified.

## Data Availability Statement

The raw data supporting the conclusions of this article will be made available by the authors, without undue reservation.

## Ethics Statement

The studies involving human participants were reviewed and approved by Institutional Review Board of Second Hospital of Tianjin Medical University. Informed consent requirement was waived due to the retrospective nature of the study.

## Author Contributions

HF and TL: conception of the work. HCho and HChe: collection, analysis data, and drafting of manuscript. JX: statistical method guidance. AX, GM, and FH: recorded and analyzed data. GL and GT: data interpretation and critically revised the manuscript. All authors contributed to the article and approved the submitted version.

## Conflict of Interest

The authors declare that the research was conducted in the absence of any commercial or financial relationships that could be construed as a potential conflict of interest.
